# Negative-Pressure Wound Therapy in a* Pseudomonas aeruginosa* Infection Model

**DOI:** 10.1155/2018/9496183

**Published:** 2018-05-15

**Authors:** Guoqi Wang, Zhirui Li, Tongtong Li, Song Wang, Lihai Zhang, Licheng Zhang, Peifu Tang

**Affiliations:** ^1^Department of Orthopedics, Chinese PLA General Hospital, No. 28 Fuxing Road, Beijing 100853, China; ^2^Department of Orthopedics, Tianjin Hospital, No. 406 Jiefangnan Road, Tianjin 300211, China; ^3^Medical College, Nankai University, Tianjin 300071, China

## Abstract

**Background:**

Negative-pressure wound therapy (NPWT) is an effective strategy for the management of contaminated wounds, including those infected by* Pseudomonas aeruginosa*. We hypothesized that NPWT would reduce virulence factors as well as biofilm components and inhibit virulence-regulated gene expression in a model of* P. aeruginosa *wound infection.

**Methods:**

Wounds were created in anesthetized rabbits and* P. aeruginosa *was inoculated to the wound surface for 24 h. Wounds were treated with either NPWT or a sterile gauze dressing. Virulence factors including exotoxin A, rhamnolipid, and elastase were quantified by the enzyme-linked immunosorbent assay, orcinol, and elastin-Congo red methods, respectively. A biofilm component, eDNA, was quantified using a commercial kit. Virulence-regulated genes were determined by quantitative real-time polymerase chain reaction (RT-PCR). Biofilms were observed* in vivo* by staining with concanavalin A conjugated to Alexa Fluor® 647.

**Results:**

NPWT was more effective than the control treatment in reducing virulence factors and bacteria counts* in vivo*. A biofilm component, eDNA, was less abundant in the NPWT group. The results of the RT-PCR indicated that the expression levels of* P. aeruginosa* virulence-regulated genes and quorum-sensing population density-dependent systems were significantly inhibited by NPWT treatment.

**Conclusion:**

NPWT reduced bacteria counts, virulence factors, and eDNA in a* P. aeruginosa* wound infection model* in vivo*. These beneficial effects are likely to be related to the reduced expression of virulence-regulated genes and the drainage induced by NPWT treatment. These findings may help clinicians to obtain a better understanding of the mechanism of NPWT for the treatment of infected wounds.

## 1. Introduction

Infections involving biofilm formation have been considered to be one of the most difficult problems in wound care for a long time [1–4]. As a common opportunistic pathogen,* Pseudomonas aeruginosa* has been studied widely, especially regarding its biofilm formation as a mechanism of infection [5–7]. Many studies have demonstrated that biofilm formation by* P. aeruginosa* is a key factor for aggravating the inflammatory response in skin and impairing wound healing [5–7]. Some virulence determinants of* P. aeruginosa* including exotoxin A, rhamnolipid, and elastase have been identified and the production of these determinants has been postulated to contribute to the failure of* P. aeruginosa*-infected wounds to heal [8–11].

A considerable number of studies have focused on the treatment of wounds infected by biofilm-forming* P. aeruginosa*, and several methods have been developed for the removal of biofilms and virulence factors. As a traditional therapy, serial debridement and lavage can remove most biofilms, virulence factors, and necrotic tissue; however, remnant bacteria may rapidly reestablish a new biofilm architecture and these treatment processes cause pain to the patients [5, 12]. Wet-to-dry treatment keeps the wound moist and helps to remove wound secretions, but it is not particularly effective in clearing* P. aeruginosa *from the wound [13]. Although some recently designed dressings for wound care have an inhibitive impact on biofilm formation, their efficacy varies greatly depending on the type, concentration, and release kinetics of the active compound [14, 15]. In addition, some biological agents, such as monoclonal antibodies, have been used for targeting bacterial biofilms. However, the clinical efficacy and safety of these compounds have not yet been sufficiently evaluated [14, 16].

Negative-pressure wound therapy (NPWT) is a medical device that has revolutionized the treatment of complex wounds over the last 20 years. As an effective management strategy for contaminated wounds, NPWT, has been widely used in clinical laboratories [17–19]. Most of the previous studies on NPWT have concentrated on the benefits of its secondary effects, including decreased edema, the removal of wound exudates, the improvement of inflammation, and the regulation of wound healing signaling pathways [20, 21]. However, investigations regarding the influence of NPWT on virulence factors and biofilm remain sparse. Previously, we demonstrated that the negative pressure induced by NPWT could influence bacterial biofilm formation and secreted factors* in vitro* [22–24]. In the present study, we aimed to evaluate the potential effect of NPWT on* P. aeruginosa* virulence factors and biofilm formation* in vivo*.

## 2. Materials and Methods

### 2.1. Animals

All animal experiments were approved by the Medical Ethics Committee of the Chinese People's Liberation Army General Hospital (Beijing, China). Japanese large-ear white rabbits (aged 3–6 months and weighing approximately 3 kg) were acclimated to standard housing and fed ad libitum under a constant temperature (22°C) and humidity (45%) with a 12 h light/dark cycle. To complete this study, a total of 22 rabbits were used.

### 2.2. Bacterial Strains and Culture

The* P. aeruginosa *wild-type strain PAO1, which carries the gene encoding green fluorescent protein (GFP), was obtained from the laboratory of the Chinese PLA Institute for Disease Control and Prevention (Beijing, China).* P. aeruginosa* was grown overnight at 37°C and subcultured in Luria–Bertani (LB) broth (AOBOX Biotechnology Co., Ltd., Beijing, China) at 37°C until the log-phase of growth was achieved. Cells were harvested by centrifugation at 4°C (5,000 ×g) and washed three times with phosphate-buffered saline (PBS). The optical density at 600 nm was measured. An optical density of 1 was equivalent to 10^5^ colony-forming units per microliter as determined by a standard curve.

### 2.3. Wounding Protocol and Bacterial Biofilm Model

The wound model used in this study was adapted from the principles established in our previously published* in vivo* study, with minor modifications [25]. Three days prior to the surgical procedure, the backs of the animals were shaved with a standard electric shaving machine. A depilatory paste was used to obtain hairless skin. All animals were anaesthetized by intramuscular injection with a mixture of ketamine (45 mg/kg; Gutian Pharma Co., Ltd., Fujian, China) and xylazine (5 mg/kg; Huamu Animal Health Product Co., Ltd., Jilin, China). Following the intradermal injection of 1% lidocaine, bilaterally symmetrical standardized 2.5 cm diameter full-thickness circular segments were excised beside the spine in the middle of the back from an area prepared with povidone-iodine solution. The tissue was excised down to the deep fascia and a wound area of approximately 5 cm^2^ was created on each side. All the wounds were inoculated with 0.3 mL of 10^8^ colony-forming units/mL of* P. aeruginosa* and dressed with semiocclusive IV3000 transparent adhesive film dressing (Smith & Nephew Healthcare Ltd., Hull, UK) for 24 h to create a bacterial biofilm model.

### 2.4. Treatment and Wound Samples Harvesting

The bilateral wounds on each rabbit were randomly assigned to two groups. Wounds in NPWT group were treated by NPWT (Weisidi Medical Science and Technology Co., Ltd., Wuhan, China) and wounds in the control were treated by sterile gauze dressing. Condition of NPWT group was continuous suction with a negative pressure of 125 mmHg. Dressings in both groups were checked daily and changed every 48 h.

Wound samples were collected according to a previously established method [25] at day 0 (24 h after bacterial inoculation) and day 2, day 4, day 6, and day 8. Biopsies were obtained from the wound center using a scalpel and then stored in sterile centrifuge tubes at 4°C.

### 2.5. Detection of Virulence Factors and eDNA

The dorsal side of each sample was removed to eliminate the possible interference of other bacteria outside of the wound surface. Samples were homogenized in tissue grinder with 1 mL sterile PBS and sonicated to remove bacterial biofilms from the tissue for 2 min in centrifuge tubes. Then, the samples were centrifuged (13,400 ×g at 4°C) to remove insoluble substances. The supernatant was used for the detection of virulence factor gene expression.

Exotoxin A was measured according to the method of Shigematsu et al. [26] and was determined using a commercially available human* Pseudomonas* exotoxin A enzyme-linked immunosorbent assay kit (Cusabio Biotech Co., Ltd., Hubei, China), according to the manufacturer's instructions. The data were recorded as ng/mL.

Rhamnolipid was quantified by the orcinol method as previously described with a few modifications [27]. Briefly, 400 *μ*L supernatant was extracted twice using 600 *μ*L diethyl ether. The ether layer was transferred to a fresh tube for evaporation. Residues were dissolved in a solution of 150 *μ*L H_2_O, 100 *μ*L 1.6% orcinol (Sigma-Aldrich, St. Louis, MO, USA), and 750 *μ*L 60% sulfuric acid (H_2_SO_4_). After heating for 30 min at 80°C, all the tubes were cooled at room temperature for 30 min and their optical absorbance was recorded at 421 nm. The concentrations of rhamnolipid were calculated by multiplying rhamnose values by a coefficient of 2.5, as previously described [28].

The activity of elastase was measured by the elastin-Congo red assay, as previously described [27]. Briefly, 100 *μ*L supernatant was added to tubes containing 10 mg elastin-Congo red (Sigma-Aldrich) and 900 *μ*L Na_2_HPO_4_ (pH 7). Tubes were incubated for 4 h at 37°C under shaking conditions and the absorbance was recorded at 495 nm after removing the precipitate by centrifugation.

For the determination of eDNA concentrations, small portions of the NPWT sponge and gauze were extracted, weighed, and then placed into centrifuge tubes with 1 mL PBS and sonicated for 2 min. After centrifugation (13,400 ×g at 4°C), the supernatant was used for eDNA detection. The TIANamp micro DNA kit (Tiangen Biotech Co., Ltd., Beijing, China) was used to extract eDNA according to the manufacturer's protocol [29]. The eDNA level was expressed as the concentration of the isolated DNA quantified using a Qubit® 2.0 Fluorometer with the Qubit dsDNA BR assay kit (Life Technologies, Carlsbad, CA, USA) according to the manufacturer's instructions. Data were recorded as *μ*g/mL/g of dressing.

### 2.6. Total mRNA Extraction and RT-PCR Analysis

A tissue specimen weighing 300 mg was removed from the erector spinae muscle of each animal. Using TRIzol® (Thermo Fisher Scientific, Waltham, MA, USA) according to the manufacturer's protocol, the supernatant was harvested for RNA extraction and the pellet was resuspended in 250 *μ*g/mL lysostaphin (Omega BioTek, Norcross, GA, USA), incubated at 37°C for 15 min, and then used for the bacterial RNA extraction. RNA was reverse-transcribed into cDNA using the TIANScript RT kit (Tiangen Biotech). For the quantitative analysis of the expression level of mRNAs, RT-PCR analyses using SYBR® FAST universal qPCR master mix (2x) (Roche, Basel, Switzerland) were performed with an ABI7900HT sequence detection system (Applied Biosystems, Foster City, CA, USA). The cycling conditions were as follows: one cycle at 95°C for 3 min; 40 cycles at 95°C for 3 s and 60°C for 20 s; and one dissociation step at 95°C for 15 s, 60°C for 15 s, and 95°C for 15 s. The primers used to amplify* toxA*,* rhlA*,* lasB*,* lasI*, and* rhlI*, as well as the reference gene,* rpoD*, are shown in Table 1. All samples were analyzed in triplicate and normalized against the expression of* rpoD*. Results were shown as the fold-change of gene expression relative to the control.

### 2.7. Viable Bacterial Counts

Samples were excised as described above in the protocol for the measurement of virulence factor gene expression. Tissue samples were homogenized in tissue grinder with 1 mL sterile PBS and then sonicated for 2 min to disrupt the biofilm in centrifuge tubes. Standard colony counting methods were used to determine the number of colony-forming units [5, 29].

### 2.8. Imaging Aggregates in Wound Sections

Samples were embedded in optimal cutting temperature compound (Sakura Finetek USA, Inc., Torrance, CA, USA) and quickly frozen, and tissue sections were obtained using a CM1950 freezing microtome (Leica Microsystems GmbH, Wetzlar, Germany) at day 2. The* P. aeruginosa *glycocalyx was visualized by staining tissue sections with 150 *μ*g/mL of concanavalin A and Alexa Fluor 647 conjugate (Thermo Fisher Scientific) for 15 min in the dark at room temperature. The sections were then washed three times with PBS and incubated with DAPI (4′,6′-diamidino-2-phenylindole dilactate, Thermo Fisher Scientific) to visualize the host cells [6, 30]. A BX51 microscope (Olympus Corporation, Tokyo, Japan) was used to visualize the fluorescence emission of each dye.

### 2.9. Statistical Analysis

Data are presented in graphic form as the mean ± standard deviation when applicable. Serial changes of virulence gene expression and virulence factors analysis were compared using a two-way analysis of variance with repeated measures, followed by a paired multivariate analysis of variance to test multiple pairwise comparisons. Bonferroni's significant difference tests were performed to analyze changes over time in within-subject characteristics. All statistical analyses were performed using SPSS 19.0 software (IBM, Armonk, NY, USA). The level of significance was set at *p* < 0.05.

## 3. Results

### 3.1. Detection of Virulence Factors and eDNA

According to a previously established wound model,* P. aeruginosa*-infected wounds were treated with NPWT or gauze from day 0. The contents of exotoxin A, rhamnolipid, and elastase secreted by* P. aeruginosa* in the tissue were measured to compare the effects of NPWT and gauze treatment on the main virulence factors.

**Figure 1:**
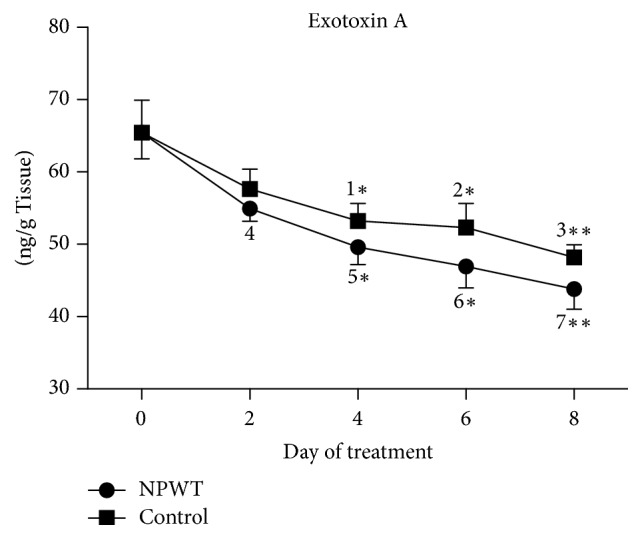
The concentration of exotoxin A in the* Pseudomonas aeruginosa*-infected wound samples significantly differed between the control group (treated with a gauze dressing) and the negative-pressure wound therapy- (NPWT-) treated group at days 4, 6, and 8 (days 4 and 6, *p* < 0.05; day 8, *p* < 0.01). In the control group, the concentration of exotoxin A significantly decreased at days 4 (*p* < 0.01 versus days 0 and 2), 6 (*p* < 0.01 versus day 0), and 8 (*p* < 0.05 versus days 0, 2, and 4). In the NPWT group, the concentration of exotoxin A significantly decreased at days 2 (*p* < 0.01 versus day 0), 4 (*p* < 0.05 versus days 0 and 2), 6 (*p* < 0.05 versus days 0, 2, and 4), and 8 (*p* < 0.01 versus days 0, 2, 4, and 6). ^*∗∗*^*p* < 0.01. Data are presented as mean ± standard deviation (*n* = 6). NPWT, negative-pressure wound therapy.

The concentration of exotoxin A decreased over time in both experimental groups. Significant decreases in the concentration of exotoxin A were observed at day 2, day 4, day 6, and day 8 compared with day 0 (Figure 1, versus day 2, *p* < 0.01; versus days 4, 6, and 8, *p* < 0.001) in the NPWT group. In the control group, a significant decrease in the concentration of exotoxin A was observed at days 4, 6, and 8 compared with day 0 (versus days 4 and 6, *p* < 0.01; versus day 8, *p* < 0.001). The NPWT group showed a trend towards a lower concentration of exotoxin A from day 2, and significant differences were observed at days 4, 6, and 8 (days 4 and 6, *p* < 0.05; day 8, *p* < 0.01).

**Figure 2:**
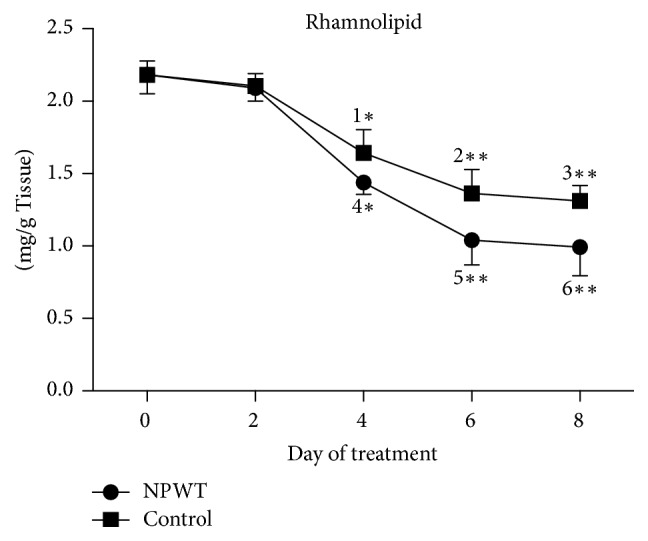
The concentration of rhamnolipid significantly differed between the two groups at days 4, 6, and 8 (day 4, *p* < 0.05; days 6 and 8, *p* < 0.01). In the control group, the concentration of rhamnolipid significantly decreased at days 4 (*p* < 0.05 versus days 0 and 2), 6 (*p* < 0.01 versus days 0 and 2), and 8 (*p* < 0.001 versus days 0 and 2). In the NPWT group, the concentration of rhamnolipid significantly decreased at days 4 (*p* < 0.001 versus days 0 and 2), 6 (*p* < 0.01 versus days 0, 2, and 4), and 8 (*p* < 0.01 versus days 0, 2, and 4). ^*∗∗*^*p* < 0.01. Data are presented as mean ± standard deviation (*n* = 6). NPWT, negative-pressure wound therapy.

The concentration of rhamnolipid also decreased over time in both groups. The extent of the decrease in the concentration of rhamnolipid did not significantly differ between the two groups (*p* = 0.784) at day 2, and significant differences were observed at days 4, 6, and 8 (Figure 2, day 4, *p* < 0.05; days 6 and 8, *p* < 0.01). The concentration of rhamnolipid was significantly higher at day 4 (Figure 2, *p* < 0.01) than at day 0 in both groups and was significantly lower at day 8 as compared with days 2, 4, and 6 in the NPWT group (versus day 2, *p* < 0.001; versus days 4 and 6, *p* < 0.05). Although a trend towards a decrease in the concentration of rhamnolipid was also observed at day 8 in the control group, no significant difference was observed in comparison to days 2, 4, and 6.

**Figure 3:**
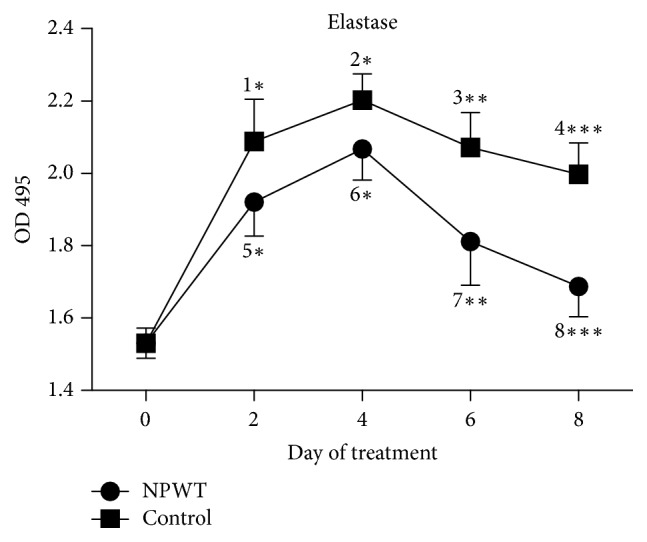
The abundance of elastase significantly differed between the two groups at days 2, 4, 6, and 8 (*p* < 0.05). In the control group, the abundance of elastase significantly increased at days 2 (*p* < 0.001 versus day 0), 4 (*p* < 0.001 versus day 0), 6 (*p* < 0.01 versus day 0), and 8 (*p* < 0.001 versus day 0). In the NPWT group, the abundance of elastase significantly increased at days 2 (*p* < 0.001 versus day 0), 4 (*p* < 0.01 versus day 0), 6 (*p* < 0.01 versus day 0), and 8 (*p* < 0.01 versus day 0) as compared with day 0. The abundance of elastase significantly decreased at day 8 (*p* < 0.05 versus days 2, 4, and 6). ^*∗*^*p* < 0.05, ^*∗∗*^*p* < 0.01, and ^*∗∗∗*^*p* < 0.001. Data are presented as mean ± standard deviation (*n* = 6). NPWT, negative-pressure wound therapy.

The abundance of elastase at day 8 was higher than that at day 0 in both groups (NPWT, *p* < 0.01; control, *p* < 0.001). The abundance of elastase in the NPWT group was significantly lower than that in the control group from days 2 to 8 (Figure 3, days 2 and 4, *p* < 0.05; day 6, *p* < 0.01; day 8, *p* < 0.001).

**Figure 4:**
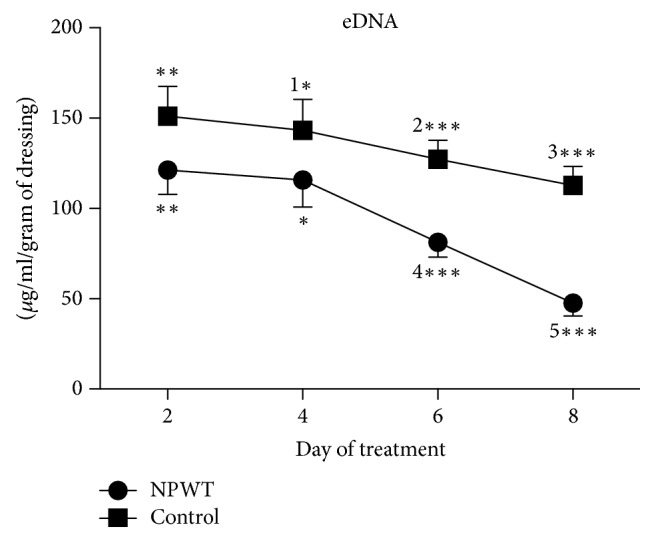
The concentration of eDNA significantly differed between the two groups at days 2, 4, 6, and 8 (day 2, *p* < 0.01; day 4, *p* < 0.05; days 6 and 8, *p* < 0.001). In the control group, the concentration of eDNA significantly decreased at days 4 (*p* < 0.05 versus day 2), 6 (*p* < 0.05 versus day 2), and 8 (*p* < 0.01 versus days 2, 4, and 6). In the NPWT group, the concentration of eDNA significantly decreased at days 6 (*p* < 0.01 versus days 2 and 4) and 8 (*p* < 0.01 versus days 2, 4, and 6). ^*∗*^*p* < 0.05, ^*∗∗*^*p* < 0.01, and ^*∗∗∗*^*p* < 0.001. Data are presented as mean ± standard deviation (*n* = 6). NPWT, negative-pressure wound therapy; eDNA, extracellular DNA.

The concentration of eDNA showed a tendency towards a decrease from days 4–8 and significant differences were observed in the NPWT group at days 6 and 8 as compared with day 2 (*p* < 0.01). In the control group, significant differences were observed at days 4, 6, and 8 as compared with day 2 (days 4 and 6, *p* < 0.05; day 8, *p* < 0.01). The NPWT group showed a significantly lower concentration of eDNA than the control group did at days 2, 4, 6, and 8 (Figure 4, day 2, *p* < 0.01; day 4, *p* < 0.05; days 6 and 8, *p* < 0.001).

### 3.2. Gene Expression

**Figure 5:**
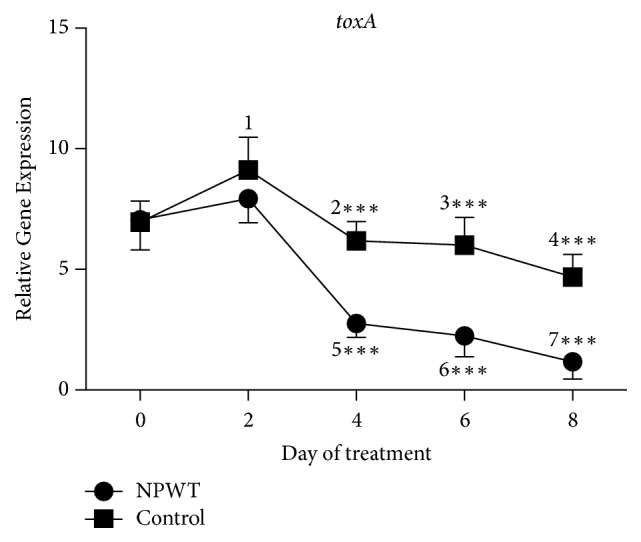
The expression of* toxA* significantly differed between the two groups at days 4, 6, and 8 (*p* < 0.001). In the control group, the expression of* toxA* significantly increased at day 2 (*p* < 0.05 versus day 0) and significantly decreased at days 4 (*p* < 0.05 versus day 2), 6 (*p* < 0.05 versus day 2), and 8 (*p* < 0.05 versus days 0, 2, and 4). In the NPWT group, the expression of* toxA* increased at day 2 (*p* > 0.05 versus day 0) and significantly decreased at days 4 (*p* < 0.001 versus days 0 and 2), 6 (*p* < 0.01 versus days 0 and 2), and 8 (*p* < 0.01 versus days 0, 2, 4, and 6). ^*∗∗∗*^*p* < 0.001. Data are presented as mean ± standard deviation (*n* = 9). NPWT, negative-pressure wound therapy.

An increase in the expression of* toxA* was observed at day 2 in both groups, but the increase was only significant in the control group (Figure 5, *p* < 0.05). The expression of* toxA* then decreased at days 4, 6, and 8 as compared with day 0 (*p* < 0.05). However, the expression of* toxA* in the control group was significantly higher than that in the NPWT group at days 4, 6, and 8 (*p* < 0.001).

**Figure 6:**
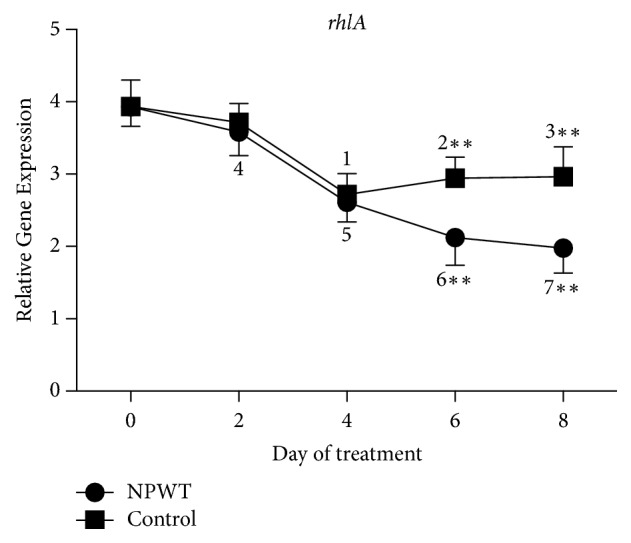
The expression of* rhlA* significantly differed between the two groups at days 6 and 8 (*p* < 0.05). In the control group, the expression of* rhlA* decreased at day 2 (*p* > 0.05 versus day 0) and significantly decreased at days 4 (*p* < 0.01 versus days 0 and 2), 6 (*p* < 0.05 versus days 0 and 2), and 8 (*p* < 0.05 versus days 0 and 2). In the NPWT group, the expression of* rhlA* significantly decreased at days 2 (*p* < 0.01 versus day 0), 4 (*p* < 0.01 versus days 0 and 2), 6 (*p* < 0.001 versus days 0 and 2), and 8 (*p* < 0.01 versus days 0, 2, and 4). ^*∗∗*^*p* < 0.01. Data are presented as mean ± standard deviation (*n* = 9). NPWT, negative-pressure wound therapy.

A significant decrease in the expression of* rhlA* was observed in both groups at days 2, 4, 6, and 8 as compared with day 0, except in the control group at day 2 (Figure 6, *p* < 0.05). The expression of* rhlA* in the control group was significantly higher than that in the NPWT group at days 6 and 8 (*p* < 0.01).

**Figure 7:**
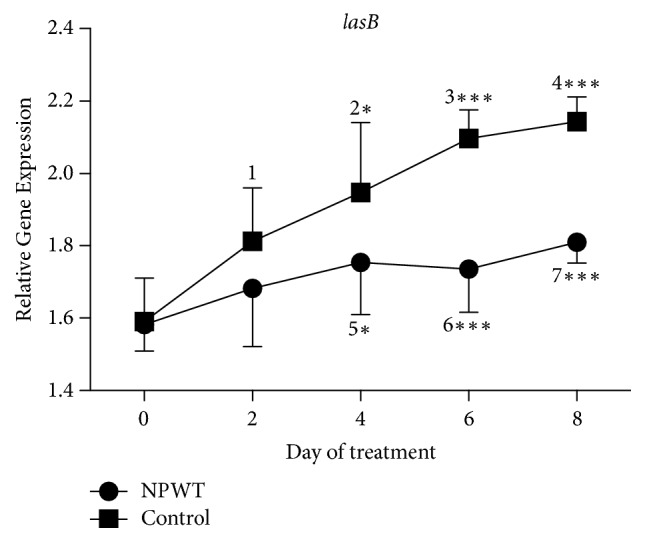
The expression of* lasB* significantly differed between the two groups at days 4, 6, and 8 (day 4, *p* < 0.05; days 6 and 8, *p* < 0.001). In the control group, the expression of* lasB* significantly increased at days 2 (*p* < 0.05 versus day 0), 4 (*p* < 0.05 versus days 0 and 2), 6 (*p* < 0.001 versus days 0 and 2), and 8 (*p* < 0.001 versus days 0 and 2). In the NPWT group, the expression of* lasB* significantly increased at days 4, 6, and 8 (*p* < 0.05 versus day 0). ^*∗*^*p* < 0.05 and ^*∗∗∗*^*p* < 0.001. Data are presented as mean ± standard deviation (*n* = 9). NPWT, negative-pressure wound therapy.

A significant increase in the expression of* lasB* was observed in both groups at days 2, 4, 6, and 8 as compared with day 0, except in the NPWT group at day 2 (Figure 7, *p* < 0.05). The expression of* lasB* in the control group was significantly higher than that in the NPWT group at days 4, 6, and 8 (day 4, *p* < 0.05; day 6, *p* < 0.001; day 8, *p* < 0.001).

**Figure 8:**
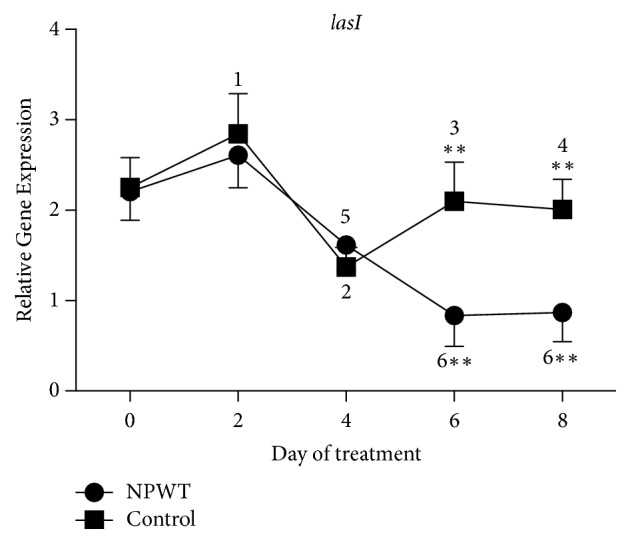
The expression of* lasI* significantly differed between the two groups at days 6 and 8 (*p* < 0.05). In the control group, the expression of* lasI *significantly increased at day 2 (*p* < 0.05 versus day 0), decreased at day 4 (*p* < 0.01 versus days 0 and 2), and then increased again at days 6 and 8 (*p* < 0.05 versus day 4), although it remained lower at days 6 and 8 than at day 0 (*p* < 0.05 versus day 0). In the NPWT group, the expression of* lasI* increased at day 2 (*p* > 0.05 versus day 0) and significantly decreased at days 4 (*p* < 0.01 versus days 0 and 2), 6, and 8 (*p* < 0.001 versus days 0, 2, and 4). ^*∗∗*^*p* < 0.01. Data are presented as mean ± standard deviation (*n* = 9). NPWT, negative-pressure wound therapy.

The expression of* lasI* in the control group was significantly increased at day 2 (Figure 8, *p* < 0.05 versus day 0), decreased at day 4 (*p* < 0.01 versus days 0 and 2), and increased at days 6 and 8 (*p* < 0.05 versus days 2 and 4). Significant decreases in the expression of* lasI* were observed at days 4 (*p* < 0.01 versus days 0 and 2), 6, and 8 (*p* < 0.001 versus days 0, 2, and 4) in the NPWT group. The differences in gene expression between the two groups were significant at days 6 and 8 (*p* < 0.05).

**Figure 9:**
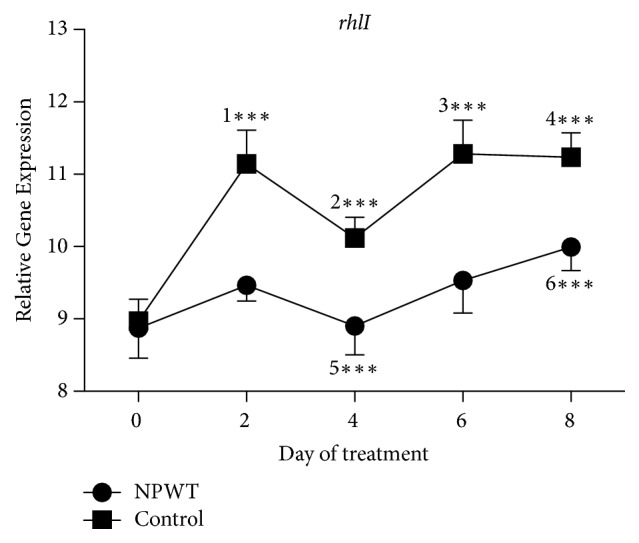
The expression of* rhlI* significantly differed between the two groups at days 2, 4, 6, and 8 (*p* < 0.001). In the control group, the expression of* rhlI* significantly increased at days 2, 4, 6, and 8 as compared with day 0 (*p* < 0.001). A significant decrease in the expression of* rhlI* was observed at day 4 (*p* < 0.001) as compared with day 2, while it significantly increased at days 6 and 8 (*p* < 0.01 versus days 0 and 4). In the NPWT group, the expression of* rhlI* increased at day 2 (*p* > 0.05 versus day 0), significantly decreased at day 4 (*p* < 0.05 versus day 2), and significantly increased at day 8 (*p* < 0.05 versus days 0, 2, and 4). ^*∗∗∗*^*p* < 0.001. Data are presented as mean ± standard deviation (*n* = 9). NPWT, negative-pressure wound therapy.

The expression of* rhlI* in the control group was significantly increased from days 2 to 8 as compared with day 0 (Figure 9, *p* < 0.001) and that in the NPWT group was significantly increased at day 8 as compared with day 0 (*p* < 0.01). The expression of* rhlI* in the control group was significantly higher than that in the NPWT group at days 2, 4, 6, and 8 (*p* < 0.001).

### 3.3. Viable Bacterial Counts

**Figure 10:**
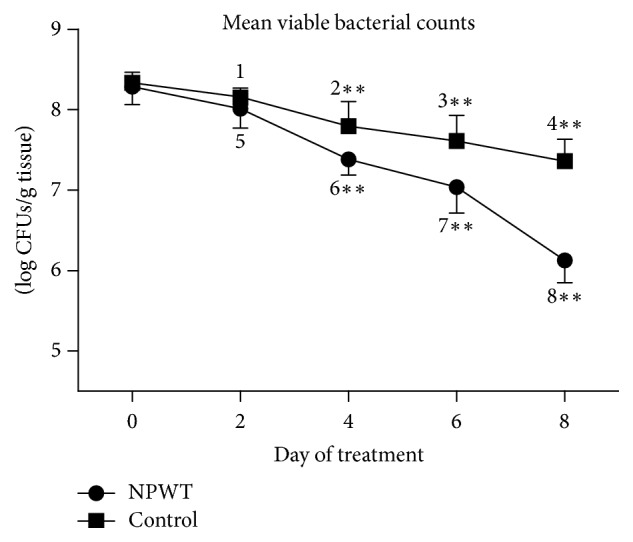
The number of viable bacteria significantly differed between the two groups at days 4, 6, and 8 (*p* < 0.01). In the control group, the viable bacterial count significantly decreased at days 2 (*p* < 0.05 versus day 0), 4 (*p* < 0.05 versus day 0), 6 (*p* < 0.01 versus days 0 and 2), and 8 (*p* < 0.05 versus days 0, 2, 4, and 6). In the NPWT group, the viable bacterial count significantly decreased at days 2 (*p* < 0.01 versus day 0), 4 (*p* < 0.001 versus days 0 and 2), 6 (*p* < 0.01 versus days 0 and 2), and 8 (*p* < 0.001 versus days 0, 2, 4, and 6). ^*∗∗*^*p* < 0.01. Data are presented as mean ± standard deviation (*n* = 6). NPWT, negative-pressure wound therapy.

The bacterial load in each group was estimated by determining the number of viable bacteria at different times. The bacterial burden in the NPWT group was not significantly different from that in the control group on days 0 and 2, although a trend towards a lower bacterial burden was observed in the NPWT group on day 2. The NPWT group showed significantly fewer viable bacteria at days 4, 6, and 8 (Figure 10, *p* < 0.01) as compared with the control group.

### 3.4. Imaging Aggregates in Wound Sections

**Figure 11:**
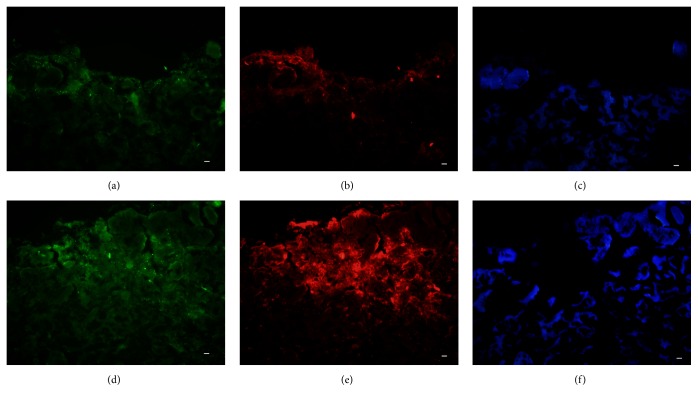
Fluorescence light microscopy of the NPWT-treated and control* P. aeruginosa*-infected wounds counterstained with ConA and DAPI at day 2. (a–c) GFP-labeled* P. aeruginosa* (green) in the NPWT group distributed over the wound (blue) with a sparse glycocalyx matrix, although large aggregates of* P. aeruginosa* are visible. (d–f) GFP-labeled* P. aeruginosa* (green) formed a mature biofilm on the control wound surface (blue), showing a complex structure with a large amount of glycocalyx (red) around the bacteria. Scale bar = 100 *μ*m. NPWT, negative-pressure wound therapy; ConA, concanavalin A; DAPI, 4′,6-diamidino-2-phenylindole; GFP, green fluorescent protein.

The presence of biofilms in wounds has been proposed to be a key impediment to wound healing via mechanisms including the provision of protection against endogenous and exogenous antimicrobial agents, the promotion of chronic inflammation, and the inhibition of reepithelization by acting as a mechanical barrier [31, 32]. However, few of these proposed mechanisms have been tested* in vivo* under NPWT treatment. Bacteria in the NPWT group were distributed over the wound beds with regional aggregation and sparse glycocalyx formation, although the bacterial aggregates were still large (Figure 11). By comparison, large amounts of glycocalyx were observed surrounding the bacteria in the wounds of the control group (Figure 11).

## 4. Discussion

NPWT has been demonstrated to represent an effective wound management strategy and has been widely used to treat various types of wounds, including acute open wounds, burn wounds, chronic wounds, and infected wounds [33–36]. NPWT could enhance wound healing despite the presence of infection. However, many studies have indicated that infected wounds still have a high bacterial load during NPWT treatment [13, 37]. Graeme performed a systematic review and concluded that there was evidence for NPWT exhibiting species selectivity and preferentially suppressing the proliferation of nonfermentative gram-negative bacilli including* P. aeruginosa* [38]. Similar results were found in another study [39]. In addition, NPWT was observed to be more effective than wet-to-dry treatment in limiting the proliferation of* P. aeruginosa *[13]. These are possible reasons for the beneficial effect of NPWT in accelerating wound healing. However, virulence factors and biofilm formation are also key factors in* P. aeruginosa *infection and how these factors change under NPWT* in vivo* has remained unclear. The goal of our study was to utilize a bacterial biofilm model to determine whether NPWT could reduce the production of* P. aeruginosa* virulence factors in infected wounds. Moreover, we investigated the integrity of biofilms and the production of their components in infected wounds treated by either NPWT or gauze dressing.

The results of the present study indicated that the production of virulence factors, gene expression, and presence of biofilm components in* P. aeruginosa*-infected full-thickness wounds were significantly different between the NPWT and control groups at several time points. The reduced production of* P. aeruginosa* virulence factors under NPWT treatment was beneficial to the host tissue, since the presence of* P. aeruginosa* virulence factors can lead to tissue necrosis. Both exotoxin A and rhamnolipid were significantly reduced by NPWT from day 2 to day 8 as compared with the control group. This benefit may arise from the drainage induced by NPWT, which has been demonstrated to represent an effective method for the removal of excess exudates [40]. Additionally, the suppression of* toxA* and* rhlA* expression under NPWT may prevent bacteria from producing more virulence factors. The abundance of elastase increased from day 0 to day 4 and reduced from day 4 to day 8 in both experimental groups. The abundance of elastase was lower in the NPWT group as compared with the control group from day 2 to day 8. Although the abundance of elastase was relatively lower in the NPWT group, it was still higher at day 8 than at day 0 in the NPWT group. Unlike the expression of* toxA* and* rhlA,* that of* lasB* increased from day 0 to day 8 in both groups, which may explain why the production of elastase was still high at day 8.

We also observed that biofilms were much more prevalent in the control wounds, but the NPWT-treated wounds manifested sparse glycocalyx and extracellular matrix without spread dispersal. Concordantly, a previous* in vitro* study found that topical negative pressure compressed the biofilm architecture with reductions in thickness and diffusion distance [41]. The detection of another key constituent of biofilm, eDNA, further supported our hypothesis that NPWT could disrupt the formation and persistence of bacterial biofilms* in vivo* but could not completely eradicate established biofilm. These benefits may result from the drainage effect and negative pressure exerted by NPWT. Previous studies have verified the effectiveness of NPWT for wound cleaning and the removal of extracellular fluid [40]. Besides,* P. aeruginosa* expresses two types of quorum-sensing (QS) population density-dependent systems, namely,* lasI-lasR *and* rhlI-rhlR*. Both these QS systems contribute to the pathology of cutaneous wound infections and have been shown to be important for biofilm formation in wounds [42, 43]. The expression of the QS systems under NPWT was inhibited as compared with the control group, which may help to prevent further biofilm formation.

Without protection from the bacterial biofilm matrix, the bacteria may be sufficiently exposed to host immunological cells and easily eliminated by the host [44], which is consistent with the viable bacteria counts observed in the present study. The bacterial counts were high in both groups on day 2 and no significant difference was observed between the groups. However, the bacterial counts in the NPWT group were lower than those in the control group on days 4, 6, and 8.

There are limitations to our study. First, wound healing was not compared between the two experimental groups. Second, we limited our study to a single bacterial species,* P. aeruginosa*. Since most patients show mixed infections, future studies regarding infections by other biofilm-forming bacteria will be required to validate the results discussed herein. Besides, NPWT was applied alone and its effects in combination with other therapies such as irrigation or antibiofilm agents were not investigated and the control wounds should be irrigated daily, as usually done in clinical settings. NPWT alone could only inhibit the formation and persistence of biofilm but not eradicate the established biofilm completely, demonstrating the high durability of biofilm and indicating the need for persistent and aggressive therapy. In future studies, we plan to investigate the efficiency of combined treatments for the removal of bacterial biofilm.

Wound infections by biofilm-forming* P. aeruginosa* are prevalent among clinical patients. It is important to recognize the commitment required to perform effective clinical wound care for these patients. In this study, NPWT was demonstrated to represent a relatively effective therapy for reducing the expression of* P. aeruginosa* virulence factors* in vivo*. In particular, NPWT reduced the amount of biofilm matrix, with corresponding reductions in the abundance of glycocalyx and eDNA, providing an opportunity for the host's immune cells to eradicate the bacteria from the wound. A better understanding of the mechanisms by which NPWT can benefit infected wounds may help doctors to provide excellent clinical wound care.

## Figures and Tables

**Table 1:** Primer sequences for quantitative RT-PCR.

Gene	Primer	Amplicon (bp)
toxA	Forward: GCCGATCTACACCATCGAGA	94
	Reverse: CATCTCGTTGCTCTCGTGC	
rhlA	Forward: TGATCACCAAGGACGACGAG	106
	Reverse: GCCAGCAGCGTGGAGATAC	
lasB	Forward: GACCCACAAGCTGTACATGAAG	110
	Reverse: CCAGCGGATAGAACATGGTG	
lasI	Forward: ACTCAGCCGTTTCGCCAT	152
	Reverse: TCATCTTCTCCACGCCTACG	
rhlI	Forward: ATTCTGGTCCAGCCTGCAA	109
	Reverse: CTGGAGGATCACGCCGTT	
rpoD	Forward: AGAGAAGGACGACGAGGAAGAAG	193
	Reverse: GGCCAGGCCGGTGAGTTC	
